# The combination use of inclisiran and statins versus statins alone in the treatment of dyslipidemia in mainland China: a cost-effectiveness analysis

**DOI:** 10.3389/fphar.2024.1283922

**Published:** 2024-02-26

**Authors:** Wenjing Zhou, Zhuoru Liang, Xiaohuan Lou, Nansong Wang, Xinyu Liu, Ruoxi Li, Pearl Pai

**Affiliations:** ^1^ The University of Hongkong-Shenzhen Hospital, Shenzhen, Guangdong, China; ^2^ The Third Affiliated Hospital (The Affiliated Luohu Hospital) of Shenzhen University, Shenzhen, Guangdong, China; ^3^ Health Commission of Shenzhen Municipality, Shenzhen, Guangdong, China; ^4^ Department of Medicine, Li Ka Shing Faculty of Medicine, The University of Hong Kong, Pokfulam, Hong Kong SAR, China

**Keywords:** Markov model, inclisiran, statins, dyslipidemia, cost-effectiveness

## Abstract

**Objective:** Statin is well-established as a classical lipid-lowering drug, and its cost has reduced considerably in the past years. Inclisiran is a new and effective lipid-lowering drug given as a subcutaneous injection at 6-month intervals. This study aims to evaluate the cost-effectiveness of the combination use of inclisiran and statin versus statin alone for dyslipidemia in the mainland China population.

**Methods:** The Markov decision-making model was used, and the clinical data and real-world data were collected at the University of Hong Kong–Shenzhen Hospital (HKU-SZH). Patients with cardiovascular disease (CVD) and blood lipid levels above the target on statin therapy were included as the target population and analyzed for cardiovascular events, future medical expenses, and the calculation made for the total life cost, quality-adjusted life years (QALYs), and incremental cost-effectiveness ratios (ICERs). Sensitivity analysis was conducted to evaluate the influence of parameter uncertainty on the base-case analysis results.

**Results:** If inclisiran was priced at Chinese renminbi (RMB) 20,000.00 (USD 2,973.49) per injection, patients in the inclisiran and statin group would incur an incremental cost of RMB 449,233.56 (USD 66,789.60) compared with the statin group, and they would obtain 0.21 more QALYs in their life cycle. The subsequent ICER of RMB 2,127,756.78 (USD 316,343.32)/QALY was significantly higher than the willingness-to-pay (WTP) threshold of 3 times the *per capita* GDP of China, which was RMB 257,094.00 (USD 38,223.33)/QALY, suggesting that the combined use of inclisiran and statin was not cost-effective. If the price of inclisiran were reduced to RMB 2,500.00 (USD 371.69)/injection, the ICER of patients in the inclisiran and statin group would become RMB 257,790.63 (USD 38,326.91)/QALY, which is slightly lower than the WTP threshold of 3 times the *per capita* GDP of China, indicating that the combined use of inclisiran and statin would be cost-effective.

**Conclusion:** If inclisiran is priced at RMB 20,000.00 (USD 2,973.49)/injection, then the combined use of inclisiran and statins is not cost-effective compared with statin alone. It will be economical only if the price of inclisiran is reduced by more than 88%.

## 1 Introduction

Globally, the number of cardiovascular disease (CVD) patients has nearly doubled from 270 million in 1990 to 520 million in 2019, making it a major disease burden of the world (Roth et al., 2020). According to the Report on Cardiovascular Health and Diseases in China 2022, CVD has become the most prominent cause of deaths among urban and rural residents in China (The Writing Committee of the Report on Cardiovascular Health and Diseases in China, 2023). The prevalence of dyslipidemia in Chinese adults has increased significantly in recent years. According to the results of nutrition and chronic disease monitoring of Chinese residents in 2018, the overall prevalence of dyslipidemia in Chinese adults was 35.6% (National Health and Family Planning Commission National Administration of Disease Control and Prevention, 2020). It has been shown that reducing low-density lipoprotein cholesterol (LDL-C) levels can significantly reduce the risk of morbidity and death from atherosclerotic cardiovascular disease (ASCVD) (Baigent et al., 2005; Moran et al., 2010). Statins have been used as the drug of choice for lowering LDL-C levels. Since the National Healthcare Security Administration of China implemented the centralized bulk purchasing of drugs in 2018, drug prices have significantly reduced, including those of commonly used statins, such as atorvastatin, rosuvastatin, and simvastatin (National Healthcare Security Administration, 2019a; National Healthcare Security Administration, 2019b). However, in many at-risk patients, LDL-C levels remain elevated despite the use of statins (Jones et al., 2012; Barkas et al., 2015; Zhang et al., 2021). A Chinese CHILLAS cohort study showed that a 1–2-fold increase in the dose of statins did not further reduce the incidence of cardiovascular events (Zhao et al., 2014). In addition, according to the Chinese Guidelines for Lipid Management (2023) (Wang et al., 2023), Chinese people demonstrate a poorer tolerance to high-dose statins than Europeans and Americans, so high-intensity and high-dose statins are not recommended.

Proprotein convertase subtilisin–kexin type 9 (PCSK9), a serine protease, binds to the LDL receptor (LDL-R) on the surface of liver cells that degrades LDL-C, thereby increasing the level of LDL-C in blood (Lakoski et al., 2009; Mousavi et al., 2009). Inclisiran is a double-stranded, chemically synthesized small interfering RNA (siRNA) that directly antagonizes PCSK9 mRNA (Jameson et al., 2014; Stone et al., 2014) and inhibits PCSK9 synthesis. Inclisiran was approved for marketing in the European Union in December 2020 and from the US Food and Drug Administration (FDA) in December 2021. The recommended drug regimen of inclisiran is a single dose of 284 mg administered subcutaneously on day 1 and day 90, and then once every 6 months (Stone et al., 2014). Inclisiran lowers the level of LDL-C by 50% or more with infrequent injections (Fitzgerald et al., 2017; Ray et al., 2017; Sabatine et al., 2017). Kosmas et al. (2020) and Merćep et al. (2022) reported that inclisiran could improve patient compliance due to a simpler dosing regimen. Some countries, such as Australia, Switzerland, and the United States, have conducted pharmacoeconomic analyses of inclisiran (Kam et al., 2020; Desai et al., 2022; Galactionova et al., 2022). The results suggested that the cost-effectiveness of inclisiran is variable across regions due to different disease incidence, drug pricing, and overall GDP levels.

Inclisiran is not yet commercially available in mainland China. Its cost-effectiveness in Chinese people with dyslipidemia is not clear. In order to speed up the availability of life-saving drugs in China, the Chinese government passed a new policy called the “Hong Kong–Macao Drug and Medical Device Policy” (National Medical Products Administration NMPA, 2020; Guangdong Medical Products Administration, 2021), which enabled the University of Hong Kong–Shenzhen Hospital (HKU-SZH) to import certain approved drugs for patient use as a pilot program. HKU-SZH was the first hospital in China to obtain this drug in August 2022. The drug has been administered in the hospital environment under direct observation. The real-world data thus generated are being used to provide the first account of the cost-effectiveness of this drug in China. In this study, we aimed to evaluate whether the combined use of inclisiran and statin is cost-effective in the treatment of patients with dyslipidemia in China compared with statin alone.

## 2 Materials and methods

### 2.1 Characteristics of the study population and study design

In this study, we used a Markov model (Moran et al., 2010) to evaluate the cost-effectiveness of inclisiran combined with statins versus statins alone in the treatment of dyslipidemia. The treatment schemes in our study were obtained from ORION-10, a double-blind, placebo-controlled, phase III randomized clinical trial (RCT) (Ray et al., 2020). Patients in this study were divided into the statin group and inclisiran group. In the statin group, patients were given statin alone (rosuvastatin/atorvastatin 10–20 mg/day or simvastatin tablet 20–40 mg). In the inclisiran group, patients were given inclisiran with statins (inclisiran was given as a single dose of 284 mg via subcutaneous injection on day 1 and day 90, and then once every 6 months).

The characteristics of the Markov model population in our study were based on the real-world data obtained from HKU-SZH between June 2022 and June 2023. Patients who received statins at a stable dose as a lipid-lowering therapy for at least 1 month or were intolerant to statins and had a baseline LDL-C level of 70 mg/dL (1.81 mmol/L) or above were eligible for this study. A total of 27,906 patients were included in this study. Of these, 121 patients were treated with inclisiran and statins, and 27,785 patients were treated with statins alone. The baseline characteristics of the patients are shown in Table 1.

**TABLE 1:** Characteristics of patients.

Characteristics	Statin N = 27,785	Inclisiran and statin N = 121
Age, yrs (SD)	59.82 (14.54)	53.26 (10.50)
Male, n (%)	17,015 (61.24)	84 (69.42)
LDL-C, mmol/L (SD)	3.05 (1.17)	2.80 (1.46)
MI, n (%)	649 (2.34)	/^a^
Statins used		
Atorvastatin	12,618 (45.41)	
Rosuvastatin	16,277 (58.58)	
Simvastatin	3,673 (13.22)	
Fluvastatin	9 (0.03)	

^a^ The occurrence of MI in the inclisiran group cannot be observed due to the short introduction time of inclisiran and the small number of patients.

### 2.2 Model structure

**FIGURE 1:**
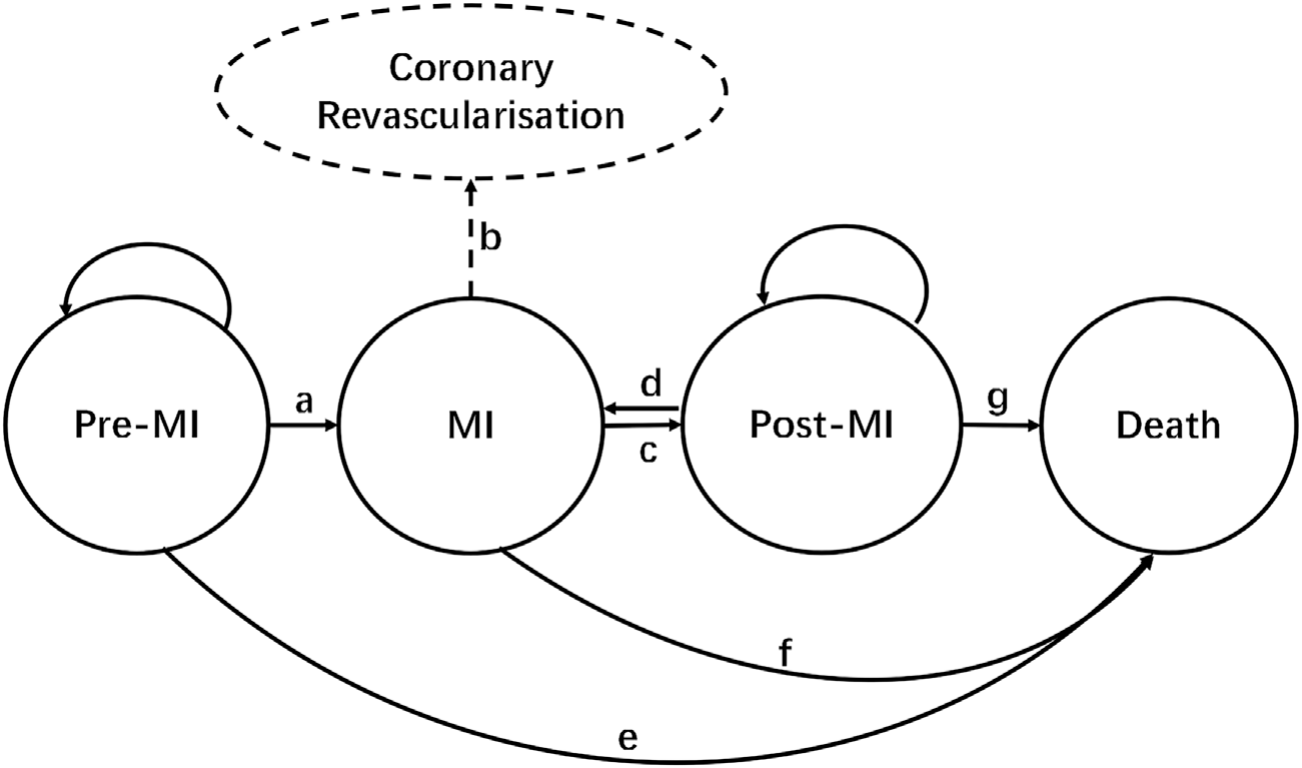
Markov model structure. Route a: MI occurs; route b: coronary revascularization; route c: transition from MI to post-MI (1 year later); route d: recurrence of MI; route e: non-CVD death; route f: death from MI; route g: death during post-MI.

According to the natural disease progression of hyperlipidemia patients with a background of CVD, the progression of the life cycle of the targeted population which received inclisiran and statins versus statins alone was assigned to four stages: (1) pre-MI, (2) MI, (3) post-MI, and (4) death. The structure of our Markov model is shown in Figure 1. In the model, patients remained healthy (pre-MI state) until they developed a MI (route a) or died of any non-CVD-related cause (route e). When a MI occurred, patients would be transferred to a MI state and might undergo coronary revascularization (route b). Patients would move to a post-MI state 1 year after the onset of MI (route c), where they might be at risk of a recurrent MI (route d). The cycle length of the Markov model was set to 1 year in this study, with the total simulation time as the life cycle of patients. TreeAge Pro 2011 software was used to build the Markov model in this study.

Route a: MI occurs; route b: coronary revascularization; route c: transition from MI to post-MI (1 year later); route d: recurrence of MI; route e: non-CVD-related death; route f: death from MI; and route g: death during post-MI.

### 2.3 Transition probability

Transition probabilities are summarized in Table 2. The annual transition probability of MI in the statin group was converted by the annual incidence rate of MI (2.34%) in patients in HKU-SZH. The annual transition probability was estimated as follows: $Annual\;transition\;probablity=1-\exp\left(\left(\ln\left(1-annual\;incidence\;rate\right)\right)/1\right)$ . For the inclisiran group, as the annual incidence rate of MI was not available from the real-world data, the annual transition probability of MI was estimated by adjusting the RR from a meta-analysis of three inclisiran clinical trials (ORION-9, ORION-10, and ORION-11), which have demonstrated a lower risk of MI in the inclisiran group than that in the control (RR = 0.68, 95% CI = 0.48–0.96) (Luo et al., 2023).

**TABLE 2:** Main assumptions for the cost-effectiveness analysis.

Parameter	Baseline value	Upper and lower limits	Distribution	Source
Event incidence rate, annual				
Incidence of MI (statin group)	2.34%	1.87%–2.81%	β distribution	In-hospital data
RR of MI (inclisiran VS statin)	0.68	0.48–0.96	β distribution	Luo et al. (2023)
Incidence of revascularization	3.2%	2.56%–3.84%	β distribution	Ray et al.(2020); Cholesterol Treatment Trialists’ CTT Collaboration et al. (2010)
MI recurrence	4.1%	3.9%–4.9%	β distribution	Song et al. (2020)
Rate of rehabilitation treatment in the post-MI state	9.96%	7.97%–11.95%	β distribution	In-hospital data
Non-CVD death	Life Table	3.36‰–5.04‰	β distribution	National Demographic Data of National Bureau of Statistics (2020)
MI death	25× non-CVD death	8.4%–12.6%	β distribution	National Demographic Data of National Bureau of Statistics (2020); Song et al. (2020)
Death during the post-MI state	2.1%	1.68%–2.52%	β distribution	Song et al. (2020)
Cost				
Inclisiran (RMB/USD)/injection	RMB 20,000.00 USD 2,973.49	RMB 16,000–24,000 USD 2,378.79–3,568.19	γ distribution	In-hospital data
Statin (RMB/USD)/tablet, daily, 20 mg/tablet	RMB 3.45 USD 0.51	RMB 2.76–4.14 USD 0.41–0.62	γ distribution	In-hospital data
MI therapeutic treatment fee (RMB/USD)/year	RMB 30,381.30 USD 4,516.93	RMB 24,305.04–36,457.56 USD 3,613.54–5,420.31	γ distribution	Statistical Information CenterNational Health and Health Commission of the People’s Republic of China (2020)
Revascularization cost (RMB/USD)/year	RMB 68,625.6 USD 10,202.88	RMB 59,400.48–82,350.72 USD 88,3,134–12,243.46	γ distribution	Statistical Information CenterNational Health and Health Commission of the People’s Republic of China (2020)
Laboratory test fee (RMB(USD)/time	RMB 211.00 USD 31.37	RMB 168.8–253.2 USD 25.10–37.64	γ distribution	In-hospital data
Rehabilitation cost (first year) (RMB/USD)/year	RMB 18,758.5 USD 2,788.91	RMB 15,006–22,510 USD 2231.01–3346.66	γ distribution	In-hospital data
Rehabilitation cost (after first year) (RMB/USD)/year	RMB 10,800 USD 1,605.69	RMB 8,540–12,960 USD 1,269.68–1,926.82	γ distribution	In-hospital data
Utility value				
Pre-MI	0.84	0.73–0.95	β distribution	Wang (2021)
Post-MI	0.80	0.69–0.91	β distribution	Wang (2021)
Recurrent MI (decrement)	0.06		β distribution	Lewis et al. (2014)
Coronary revascularization (decrement)	0.07		β distribution	van Stel et al. (2012)
Discounting	5%	0–8%		

According to a previous research study, the annual incidence rate of coronary revascularization was 3.2% (Cholesterol Treatment Trialists’ CTT Collaboration et al., 2010). In-hospital data from HKU-SZH showed that approximately 9.96% of patients who received statin therapy received rehabilitation treatment in the post-MI state. Due to the lack of data, the rehabilitation rate of the inclisiran group was assumed to be equivalent to that of the statin group. The annual recurrence rate of MI in the post-MI state was assumed to be 4.1% (Song et al., 2020).

Age-specific mortality rates for non-CVD deaths in the pre-MI state were based on the Chinese Life Table obtained from the sixth nationwide census (National Demographic Data of National Bureau of Statistics, 2020). Mortality rates of patients in the MI state and post-MI state were assumed to be 25 times that of non-CVD death and 2.1%, respectively, according to the China PEACE study (Song et al., 2020).

### 2.4 Cost data

Only direct medical costs including drug, treatment, laboratory test, and rehabilitation costs were considered in this analysis. Detailed cost data are shown in Table 2.

As inclisiran was not yet available commercially in China and was only being piloted in a few hospitals (including HKU-SZH) at the time of this study, the unit price of inclisiran per injection was estimated using the real-world data from HKU-SZH. Under the “Hong Kong–Macao Drug and Medical Device Policy” (National Medical Products Administration NMPA, 2020; Guangdong Medical Products Administration, 2021) and its regulation, the drug cost of inclisiran in HKU-SZH was marked at RMB 20,000.00 (USD 2,973.49) per injection. The daily cost of statin was an adjusted cost estimated by multiplying the adjusted percentage by the average daily cost of different types of statins (Table 3). According to the data from HKU-SZH between June 2022 and June 2023, the main types of statins taken by patients were atorvastatin (45.41%), rosuvastatin (58.58%), and simvastatin tablets (13.22%).

**TABLE 3:** Basic prices of statins.

Types of drugs	Specification	Price per box ¥; $	Price per day (20 mg) ¥; $	Manufacturer	Percentage	
					Unadjusted (%)	Adjusted (%)
Atorvastatin					45.41	38.74
Atorvastatin (provincial procurement)	10 mg*28	¥ 5.6; $ 0.83	¥ 0.4; $ 0.06	Lepu Pharmaceutical Technology Co., Ltd.		
Atorvastatin (provincial procurement)	20 mg*28	¥ 9.52; $ 1.42	¥ 0.3; $ 0.04	Lepu Pharmaceutical Technology Co., Ltd.		
Atorvastatin	20 mg*7	¥ 42.77; $ 6.36	¥ 6.1; $ 0.91	Pfizer Pharmaceutical Ltd.		
Average price per day			¥ 2.3; $ 0.34			
Rosuvastatin					58.58	49.98
Rosuvastatin	10 mg*7 tablets	¥ 38.8; $ 5.77	¥ 11.10; $ 1.65	AstraZeneca Limited (China)		
Rosuvastatin (provincial procurement)	10 mg*28	¥ 5.59; $ 0.83	¥ 0.40; $ 0.06	Zhejiang Jingxin Pharmaceutical Co., Ltd.		
Rosuvastatin	20 mg*6	¥ 23.1; $ 3.43	¥ 3.90; $ 0.58	Zhejiang Jingxin Pharmaceutical Co., Ltd.		
Average price			¥ 5.1; $ 0.76			
Simvastatin					13.22	11.28
Tablets (provincial procurement)	20 mg*28	¥ 2.96; $ 0.44	¥ 0.11; $ 0.02	Zhejiang Jingxin Pharmaceutical Co., Ltd.		
Average price			¥ 0.11; $ 0.02			
Total adjusted cost			¥ 3.45; $ 0.51			

The unadjusted percentage is the proportion of patients using a statin (atorvastatin, rosuvastatin, and simvastatin), whereas the adjusted percentage is the percentage of each statin divided by the total statin, for example, the adjusted percentage of atorvastatin = 45.41%/(45.41% + 58.58%+13.22%) = 38.74%.

Patients in the inclisiran group were required to pay an injection fee of RMB 36.00 (USD 5.35) per treatment. The cost of the laboratory test for blood lipids was RMB 211.00 (USD 31.37) for each visit. After a MI, patients required hospitalization with or without coronary revascularization. The cost of hospitalization due to MI and the cost of coronary revascularization were obtained from the China Health Statistics Yearbook 2020. Patients in the post-MI state may need rehabilitation therapy. The costs of rehabilitation were sourced from the in-hospital data of HKU-SZH.

Costs were reported in Chinese renminbi (RMB) and US dollars according to the 2022 exchange rate (USD 100.00 = RMB 672.61). All costs were converted to 2023 using the medical care component of the consumer price index (CPI).

The unadjusted percentage is the proportion of patients using a statin (atorvastatin, rosuvastatin, and simvastatin), whereas the adjusted percentage is the percentage of each statin divided by the total statin, for example, the adjusted percentage of atorvastatin = 45.41%/(45.41% + 58.58%+13.22%) = 38.74%.

### 2.5 Quality of life estimation

Health outcomes were measured using quality-adjusted life years (QALYs), a combined measure of life years with health-related utility scores, which were calculated by multiplying the length of time in a health state by the utility scores with that health state [range from 0 (equivalent to death) to 1 (equivalent to perfect health)]. Due to the lack of raw data, the utility scores were obtained from previously published literature. Patients entered the model (pre-MI) with an initial utility score of 0.84. It was assumed that the utility would decrease to 0.80 when the patient subsequently experienced a MI. Utility decrements were also applied for recurrent MI (0.06) and coronary revascularization (0.07). The details of the utility scores are shown in Table 2.

### 2.6 Cost-effectiveness outcome estimation

In this study, the outcome measures of the model included the treatment cost (RMB), QALYs, and the incremental cost-effectiveness ratios (ICERs) (RMB per QALY). All costs and clinical outcomes were calculated at an annual discount rate of 5% (China Guidelines for Pharmacoeconomic Evaluations, 2011). According to the China Guidelines for Pharmacoeconomic Evaluations (2011), the willingness-to-pay (WTP) threshold for patients was recommended as 3 times the *per capita* GDP of China. According to the data released by the State Statistics Bureau, the *per capita* GDP of China in 2022 was RMB 85,698.00 (USD 12,741.11) (https://data.stats.gov.cn/easyquery.htm?cn=C01); hence, the WTP was set at RMB 257,094.00 per QALY in this study.

### 2.7 Sensitivity analysis

The single-factor sensitivity analysis and probabilistic sensitivity analysis (PSA) were performed in this study to test the influence of the uncertainty of model parameters on the results. One-way sensitivity analysis was used to evaluate the influence of a single factor on the ICER value by simulating the results of the variation in a single factor within a defined range. The upper and lower limits of the parameter variation range were its maximum and minimum values, respectively. If the maximum and minimum values of the parameter were not available, ±20% of the baseline value might be taken as the upper and lower limits of the parameter. The PSA was conducted by 1,000 second-order Monte Carlo simulations. The cost data were assumed to be of γ distribution, and the event incidence and health utility value were assumed to be of β distribution. The parameter values and their distributions are shown in Table 2.

## 3 Results

### 3.1 Base-case and scenario analyses

Table 4 shows the results of the lifetime cost-effectiveness analyses for base-case (RMB 20,000 per injection) and simulated scenarios (inclisiran is cost-effective) using the Markov model. In base-case analysis, patients in the inclisiran and statin group had an incremental cost of RMB 449,233.56 compared with the statin-alone group and gained an additional 0.21 QALYs in their life cycle. The ICER for inclisiran and statin was RMB 2,127,756.78 per QALY, which was significantly higher than the WTP threshold of 3 times the GDP *per capita* of China, suggesting that inclisiran and statin therapy was not cost-effective in this cost setting. Inclisiran is not yet commercially available in China. Any future pricing and payment modes should take into consideration the health economics. In our scenarios analysis, if the price of inclisiran were reduced to RMB 2,500 per injection, the ICER of patients administered inclisiran and statin would work out as RMB 257,790.63 per QALY, just below the WTP threshold of 3 times the GDP *per capita* in China, suggesting that inclisiran would only be cost-effective if its price were reduced by 88%.

**TABLE 4:** Lifetime cost-effectiveness results for base-case and scenario analyses.

Result	Cost (¥/$)			Effectiveness (QALY)			ICER ¥/QALY $/QALY
	Inclisiran + statin	Statin	∆	Inclisiran + statin	Statin	∆	
Base price	¥475,999.37 $ 70,769.00	¥ 26,765.81 $ 3,979.40	¥449,233.56 $ 66,789.60	9.42	9.21	0.21	¥ 2,127,756.78 $ 316,343.32
Price reduction to ¥2,500	¥ 81,193.18 $ 12,071.36	¥ 26,765.81 $ 3,979.40	¥ 54,427.37 $ 8,091.97	9.42	9.21	0.21	¥ 257,790.63 $ 38,326.91

QALY, quality-adjusted life year; ICER, incremental cost-effectiveness ratio.

### 3.2 Sensitivity analysis

**FIGURE 2:**
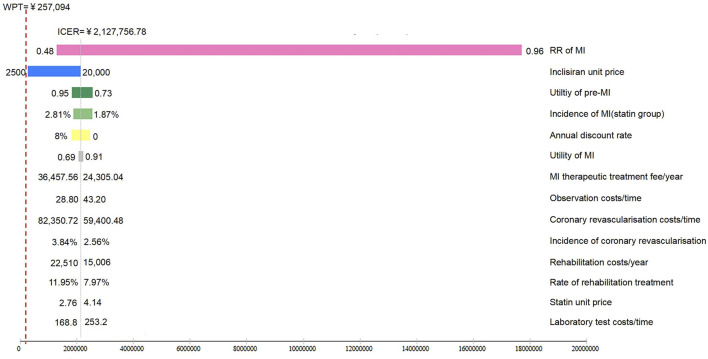
Tornado plot of inclisiran and statin versus statin alone. The tornado plot is used to demonstrate the impact of varying each of the model parameters on the ICER for inclisiran and statin versus statin alone, while the boundaries of the bar represent the ICER at the upper and lower limits of the parameters. *WTP*, willingness-to-pay.

The results of the one-way sensitivity analysis are summarized in Figure 2. The results of the Markov model simulation were most sensitive to the risk ratio of the development of MI (RR value) using inclisiran and statin versus statin therapy alone. Other sensitive parameters (in order of importance) were the unit price of inclisiran, utility of the pre-MI state (initial state), utility of the post-MI state, unit price of statin, MI hospitalization cost, and MI rehabilitation cost. The alteration of these variables within their upper and lower limits does not reverse the results of the cost-effectiveness analysis.

**FIGURE 3:**
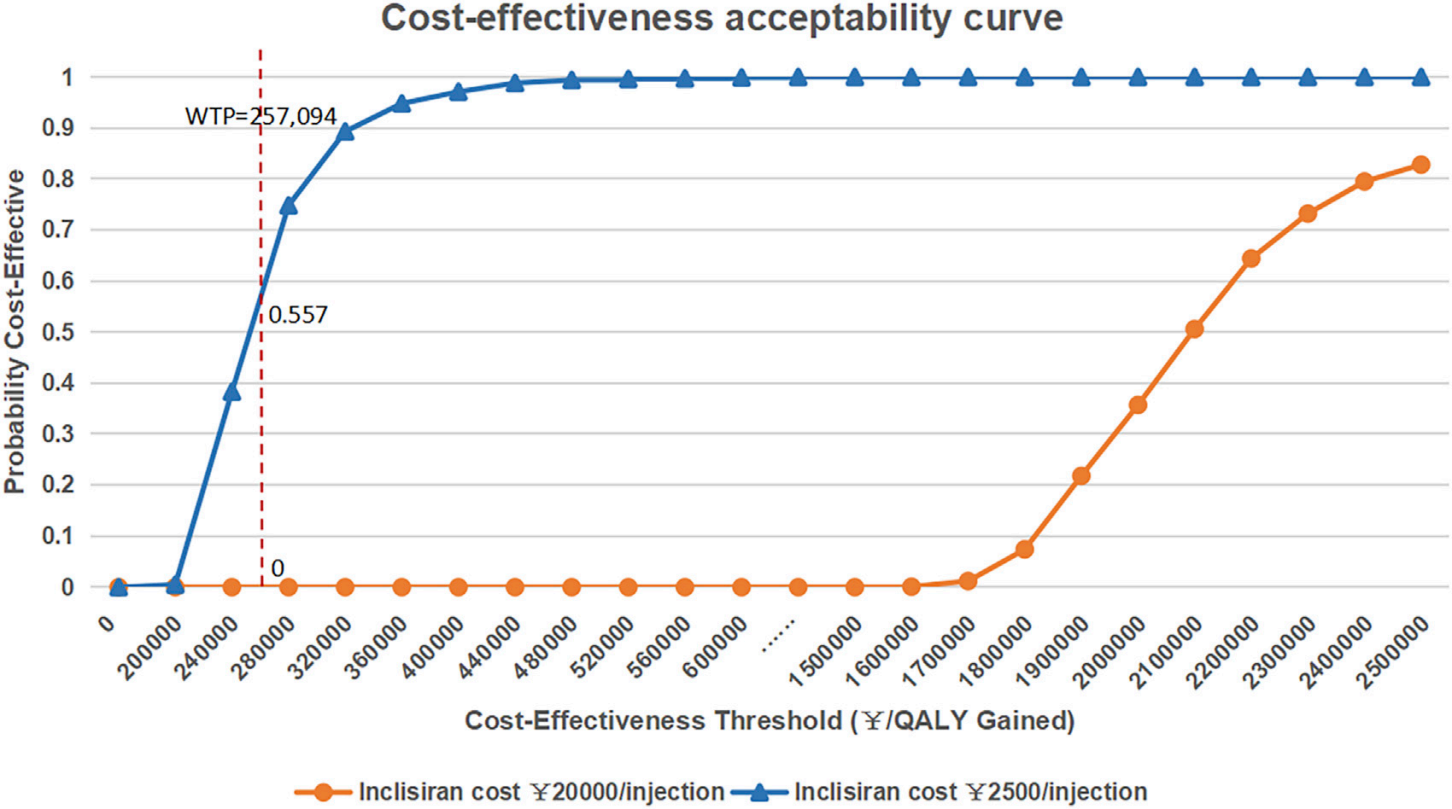
Cost-effectiveness acceptability curve of inclisiran and statin versus statin alone. *QLAY*, quality-adjusted life year; *WTP*, willingness-to-pay.

The results of the probabilistic sensitivity analysis are shown in Figure 3. The results of probabilistic sensitivity analysis show that the probability of inclisiran being cost-effective compared with statin alone was 0 at the WTP threshold of 3 times the *per capita* GDP of China (RMB 257,094), indicating that combined inclisiran and statin use was not cost-effective when inclisiran was priced at RMB 20,000 per injection. If the price of inclisiran were reduced to RMB 2,500.00/injection, the probability of inclisiran being cost-effective at the WTP threshold of 3 times the *per capita* GDP of China (RMB 257,094) was 55.7%.

## 4 Discussion

Statins are used as the standard lipid-lowering drugs for patients with dyslipidemia (Averna et al., 2021; Pearson et al., 2021; Virani et al., 2021). Inclisiran is a novel lipid-lowering drug of the PCSK9 inhibitor class with strong lipid-lowering effects and has been shown to improve patient compliance when combined with statins (Kosmas et al., 2020; Merćep et al., 2022). However, the cost-effectiveness of inclisiran in patients with dyslipidemia in different regions depends on multiple factors, such as drug pricing and level of economics.

Under the “Hong Kong–Macao Drug and Medical Device Policy” (National Medical Products Administration NMPA, 2020; Guangdong Medical Products Administration, 2021), HKU-SZH had introduced the potent lipid-lowering drug inclisiran for the first time in China as a pilot and generated relevant real-world clinical data. The cost-effectiveness of inclisiran and statins versus statin alone in patients with dyslipidemia in mainland China was analyzed for the first time in this study using real-world data including the drug costs, investigation costs, and treatment and rehabilitation costs, as well as data from the Chinese census and Health Statistical Yearbook of the respective healthcare systems.

In our study, we analyzed the cost-effectiveness of inclisiran and statins versus statin alone based on the real-world data of patients with dyslipidemia in China. The results of our study show that at RMB 20,000 per inclisiran injection, the ICER for inclisiran and statin versus statin alone was RMB 2,127,756.78 per QALY, which was significantly greater than the threshold ICER of 3 times the GDP *per capita* of China (RMB 257,094), indicating that combined inclisiran and statin therapy was not cost-effective compared with statin alone. However, if the price of inclisiran were reduced to RMB 2,500 per injection, the ICER for inclisiran and statin versus statin alone would become RMB 257,790.63/QALY, with an acceptability rate of 55.7% at a WTP threshold of 3 times the *per capita* GDP of China.

The health economics of inclisiran have similarly been studied in Australia, Switzerland, and the United States of America. Kam et al. (2020) compared the use and effect of inclisiran and statin with that of statin alone in Australian patients with dyslipidemia. The WTP in Australia was AU 50,000. As such, inclisiran was not considered cost-effective when its cost per person was AU 6,334.00 per year. In order for inclisiran to become cost-effective in the Australian healthcare system, the cost had to be reduced by 60%. Katya et al. (Galactionova et al., 2022) evaluated the cost-effectiveness of adding inclisiran to standard lipid-lowering therapy in a Swiss population with dyslipidemia. They worked out that inclisiran was cost-effective if it was priced at CHF 500.00, which yielded a WTP of CHF 30,000.00. If it were priced at CHF 3,000.00, the WTP would have to be higher than CHF 250,000.00. Nihar et al. (Desai et al., 2022) studied the use of inclisiran in addition to standard therapy in ASCVD patients in the USA compared with standard treatment alone. Using three different annual prices of inclisiran of USD 6,383.00, USD 9,973.00, and USD 13,563.00, respectively, the WTP threshold per QALY was estimated to be USD 50,000.00, USD 100,000.00, and USD 150,000.00, respectively.

### 4.1 Limitations

This study has some limitations. First, the MI event rate of patients in the statin group had been estimated from the data of patients in our hospital, whereas the rates of other events such as the MI rate in the inclisiran group were based on established RCTs and international meta-analysis studies with their own limitations. These external data may introduce variable bias due to the racial and ethnicity difference between Asian and European and USA subjects. Second, the calculation of drug cost per day was based on the average price in Guangdong Province and national bulk purchasing (Guangdong Medical Products Administration, 2021) and could be variable. Due to the differences in economic development between different regions, the drug cost in Guangdong Province could not fully represent the average level of China. To overcome these limitations, all model inputs had been evaluated over a wide range of values in the sensitivity analyses of the Markov model simulation, and the robustness of the result was confirmed. Moreover, as long as updated real-world data for inclisiran are obtained in the future, we can update our data and perform scenario analyses to re-evaluate the cost-effectiveness of inclisiran and statin versus statin alone in patients with dyslipidemia in China.

## 5 Conclusion

Our study analyzed the cost-effectiveness of inclisiran and statins versus statins alone in patients with dyslipidemia in China. Our results showed that at the current price of RMB 20,000 per inclisiran injection, the combined use of inclisiran and statin was not considered cost-effective in patients with dyslipidemia in mainland China under the present medical and economic environment. In terms of QALYs, its price would have to be reduced by more than 88% to be considered cost-effective.

## Data Availability

The raw data supporting the conclusion of this article will be made available by the authors, without undue reservation.
